# Wide distribution and altitude correlation of an archaic high-altitude-adaptive *EPAS1* haplotype in the Himalayas

**DOI:** 10.1007/s00439-016-1641-2

**Published:** 2016-02-16

**Authors:** Sophie Hackinger, Thirsa Kraaijenbrink, Yali Xue, Massimo Mezzavilla, Asan, George van Driem, Mark A. Jobling, Peter de Knijff, Chris Tyler-Smith, Qasim Ayub

**Affiliations:** The Wellcome Trust Sanger Institute, Wellcome Genome Campus, Hinxton, Cambridgeshire CB10 1SA UK; Department of Human Genetics, Leiden University Medical Center, Leiden, The Netherlands; BGI-Shenzhen, Shenzhen, 518083 China; Institute of Linguistics, University of Bern, Bern, CH 3012 Switzerland; Department of Genetics, University of Leicester, Leicester, UK; Division of Experimental Genetics, Sidra Medical and Research Center, Doha, Qatar

## Abstract

High-altitude adaptation in Tibetans is influenced by introgression of a 32.7-kb haplotype from the Denisovans, an extinct branch of archaic humans, lying within the endothelial PAS domain protein 1 (*EPAS1*), and has also been reported in Sherpa. We genotyped 19 variants in this genomic region in 1507 Eurasian individuals, including 1188 from Bhutan and Nepal residing at altitudes between 86 and 4550 m above sea level. Derived alleles for five SNPs characterizing the core Denisovan haplotype (AGGAA) were present at high frequency not only in Tibetans and Sherpa, but also among many populations from the Himalayas, showing a significant correlation with altitude (Spearman’s correlation coefficient = 0.75, *p* value 3.9 × 10^−11^). Seven East- and South-Asian 1000 Genomes Project individuals shared the Denisovan haplotype extending beyond the 32-kb region, enabling us to refine the haplotype structure and identify a candidate regulatory variant (rs370299814) that might be interacting in an additive manner with the derived G allele of rs150877473, the variant previously associated with high-altitude adaptation in Tibetans. Denisovan-derived alleles were also observed at frequencies of 3–14 % in the 1000 Genomes Project African samples. The closest African haplotype is, however, separated from the Asian high-altitude haplotype by 22 mutations whereas only three mutations, including rs150877473, separate the Asians from the Denisovan, consistent with distant shared ancestry for African and Asian haplotypes and Denisovan adaptive introgression.

**Electronic supplementary material:**

The online version of this article (doi:10.1007/s00439-016-1641-2) contains supplementary material, which is available to authorized users.

## Introduction

Genetic, biochemical and morphological changes have enabled humans to survive, adapt and reproduce in hostile high-altitude environments in Africa, Asia and South America. Among these environments, the Tibetan Plateau and the Himalayan Mountain Range in Asia is the largest and highest, and modern-day dwellers in this region include the Tibetans, Sherpa and several other populations in Bhutan and Nepal. In response to chronic hypoxia these highlanders exhibit a reduced hypoxic ventilatory response and their blood haemoglobin levels do not rise dramatically with increasing altitude, as is seen in people of low-altitude ancestry who move to high altitudes (Beall et al. 2010; Bigham and Lee 2014; Gilbert-Kawai et al. 2014).

Even though the physiological response to chronic hypoxia in Tibetans has been well documented, the evolutionary origin of this adaptive phenotype remains poorly understood. Endothelial PAS domain protein 1 (*EPAS1*), encoding a transcription factor that acts in the hypoxia-inducible factor pathway, is one of the genes that has been implicated in these physiological responses (Beall et al. 2010; Simonson et al. 2010). This pathway is the key regulator of responses to low oxygen concentration (Bigham and Lee 2014). Previous studies have identified several genetic variants within *EPAS1* that are associated with high-altitude adaptation in Tibetans and have been shown to correlate with lower haemoglobin levels in Tibetans as well as in the Nepalese Sherpa (Beall and Reichsman 1984; Samaja et al. 1979; Yi et al. 2010). One of these was rs150877473, where the derived G allele showed a 78 % higher frequency in Tibetans than in Han Chinese “representing the fastest allele frequency change observed at any human gene to date” according to the authors (Yi et al. 2010). A follow-up study showed that this single nucleotide polymorphism (SNP) lay within an extended haplotype in Tibetans, and a 32.7-kb region matched the genome of an archaic Denisovan hominin (Meyer et al. 2012), but few other modern humans (Huerta-Sanchez et al. 2014), suggesting that this haplotype might have been introduced into the Tibetan gene pool by archaic DNA introgression (Racimo et al. 2015). A parallel study compared genome-wide SNP data of Nepalese Sherpa with Tibetans and suggested that they shared a common ancestor and the high-altitude-adaptive haplotype (Jeong et al. 2014).

In the current study, we have genotyped several *EPAS1* SNPs within the introgressed intronic region in a large set of populations residing at various altitudes in or near the Himalayas. In particular, we examined additional high-altitude dwellers from Bhutan and Nepal, who are separated from the Tibetan plateau by the Himalayan mountain range, to measure the distribution of the Denisovan haplotype in these populations and to assess the haplotype structure within this region.

## Materials and methods

### DNA samples

We genotyped a total of 1969 DNA samples representing 55 populations, including 44 from Bhutan and Nepal, four from China, three from Pakistan, two from India and one each from Mongolia and the Caucasus (Table S1). The samples from Pakistan and the Adygei were from the Human Genome Diversity Project (HGDP-CEPH) cell line panel. For some analyses, we stratified the groups into three altitude categories depending upon whether they were long-term residents at high (>3000 m), medium (2000–3000 m) or low (<2000 m) altitudes above sea level (Gou et al. 2014).

Anonymized samples from Bhutan and Nepal were obtained from unrelated residents in villages located at altitudes ranging from 86 to 4550 m above sea level. These samples were collected as part of the Language and Genes of the Greater Himalayan Region Project, a genetic survey of Tibeto-Burman and Indo-European speakers from these Himalayan countries, and have been described previously (Kraaijenbrink et al. 2014). Informed consent was obtained from each individual and the study was approved by the relevant institutional review boards and covered under bilateral agreements between Leiden University Medical Center and the authorities in Nepal and Bhutan. Whole genome amplification was performed for all samples from China and for a subset of Himalayan samples using the Illustra GenomiPhi HY DNA amplification kit (GE Healthcare Life Sciences) following the manufacturer’s protocol. All except 596 Bhutanese and Nepalese samples were whole genome amplified and genomic DNA (10 ng/μl) was used for the remaining samples.

### Genotyping

We used the Sequenom mass spectrometry platform to genotype 21 of the 32 intronic *EPAS1* SNPs present at a high frequency in Tibetans in a single multiplex SNP assay. Assay Designer software (v4.0.0.2) was used to design PCR and extension primers (Table S2). We were unable to design primers for the remaining 11 of the 32 variants. Following amplification, the base extension reaction was performed on shrimp alkaline phosphatase-treated PCR products using iPLEX enzyme and mass-modified terminators as described earlier (MacArthur et al. 2012). The resulting spectra were called by the real-time SpectroCaller algorithm and analysed by SpectroTyper v.4.0 software that combines a base caller with the clustering algorithm. We were unable to call genotypes for rs369097672 and we excluded one other SNP (rs58160876) with the highest proportion (31.5 %) of missing data. We filtered out samples with discordance between reported gender and genotype sex (*n* = 79), as well as any sample with more than 20 % missing SNP calls (*n* = 380). Genotyping quality was assessed by examining concordance of SNP calls in 18 Southern Han Chinese (CHS) samples that were previously sequenced by the 1000 Genomes Project, and by comparing genotyping calls between duplicate sample pairs. For the 19 remaining SNPs the concordance with SNP calls in the 1000 Genomes Project CHS sequencing data was 97.3 %. There were 283 duplicates between Bhutanese and Nepalese whole genome-amplified and non-amplified samples, as well as three CHS samples submitted without whole genome amplification; the genotype concordance between these unamplified/amplified duplicate pairs was 98.1 %. We subsequently removed three Himalayan populations that were represented by single individuals only, and the final genotyped dataset comprised a total of 1507 samples with SNP calls for 19 intronic *EPAS1* SNPs (Table S1).

### Phasing and median-joining haplotype networks

We generated phased haplotypes for the genotyped samples using PHASE v2.1.1 (Stephens and Scheet 2005). Using the phased data, we constructed median-joining haplotype networks with the NETWORK software (v4.6.1.3) package (Bandelt et al. 1999). We also included haplotypes generated for 26 worldwide populations by the 1000 Genomes Project and from a Denisovan and a Neanderthal individual sequenced at high coverage (Meyer et al. 2012; Prüfer et al. 2014; The 1000 Genomes Project Consortium 2012). We further investigated how these were related to the archaic Denisovan haplotype by comparing mean derived allele frequencies for the genotyped SNPs across populations. The ancestral and derived states of each variant were based on a six-way primate alignment as determined by the Ensembl compara pipeline (Cunningham et al. 2015; Paten et al. 2008).

### Positive selection and haplotype structure

We estimated the positive scaled selection coefficient(*σ* = 2 × *N*_e_ × *s***)** for each genotyped SNP in *EPAS1* using SelEstim v.1.1.3 (Vitalis et al. 2014), where *N*_e_ represents the effective population size and *s* is the coefficient of selection. SelEstim is based on Wright’s island model that uses bi-allelic markers (with alleles *A* and *a*) and a diffusion approximation algorithm for the distribution of allele frequency in a population subdivided into a number of demes that exchange migrants with a ratio equal to m. For the selection model in each deme *i* at each bi-allelic locus *j*, the allele *A* offers a selective advantage. The homozygote individuals *AA* and the heterozygotes *Aa* have a relative increase of fitness of 1 + *sij* and 1 + *sij*/2, respectively, as compared to the aa homozygotes. SelEstim estimates the selection coefficients for each marker from the data. The scaled coefficient of selection in each deme i at locus j is defined as *σij* = 2*Nisij*.

To estimate *σ* we used the data from Nepalese Sherpa (SHE), Bhutanese Brokkat (KAT) and Tibetans (TIB) as proxies for high-altitude populations, and two populations each from Europe, South Asia and East Asia as proxies for low-altitude dwellers. SelEstim uses a component-wise Markov chain Monte Carlo algorithm to estimate the parameters of the selection coefficient. We used 25 short pilot runs of 1000 iterations each and a burn-in of 25,000 steps. Samples were collected for all the model parameters every 25 steps. To determine whether the best selection model was dominant, recessive or additive, we performed an association test implemented in PLINK v1.07 (Purcell et al. 2007) using population allelic frequencies of the genotyped SNPs and their residence altitudes as a quantitative phenotype (Table S1), and report *p* values for each model. In the additive model for a given bi-allelic locus with ancestral allele *a* and derived allele *A*, the fitness of the *A/A* individual is 1 + *w* whereas the fitness of the *A/a* genotype equals 1 + *w*/2, and assumes that there is no advantage of having the *a/a* genotype, i.e. the fitness of *a/a* equals 1. In the dominant model the fitness of the *A/A* and *A/a* individuals is the same (1 + *w*), whereas in the recessive model *A/A* individuals have fitness equal to 1 + *w* and *A/a* have fitness equal to *a/a*.

### Simulation of positive selection

We also performed a small-scale simulation study using simuPOP v 1.1.6 (Peng and Kimmel 2005). We simulated two populations (Han and Tibetans), using estimates of the demographic parameters—split time, expansion/reduction and effective population sizes—from the literature (Yi et al. 2010). The demographic history of the Tibetans is not well understood and the split time between the Tibetans and Han Chinese is highly debatable with estimates ranging from ~3000 to 30,000 years ago based on autosomal or uniparentally inherited markers, respectively (Qi et al. 2013; Yi et al. 2010). We simulated a locus with different initial derived allele frequencies prior to the population split (0.01, 0.05 and 0.1) and the selection coefficient for the derived allele in the Tibetans estimated by SelEstim. We used the marker with the highest scaled selection coefficient (*σ* = 2 × *N*_e_ × *s*) in Tibetans and followed its frequency across multiple generations in both Han and Tibetans for three different demographic scenarios assuming a split time of 2800, 10,000 or 30,000 years ago. Each scenario was replicated 100 times.

## Results and discussion

We obtained high-quality genotypes for 19 SNPs in a ~32-kb region within *EPAS1* on chromosome 2 in 1507 Eurasian samples representing 55 populations from seven countries (Tables S1 and S3), including 1188 samples from Bhutan and Nepal residing in villages located at altitudes ranging from 86 to 4550 m above sea level. While our primary goal was to investigate the presence of the core Denisovan haplotype in the Himalayas, we also included samples from China, India, Mongolia, Pakistan and Russia to gain a better understanding of the geographical distribution of this haplotype.

### Prevalence of the core Denisovan haplotype in the Himalayan region

**Fig. 1:**
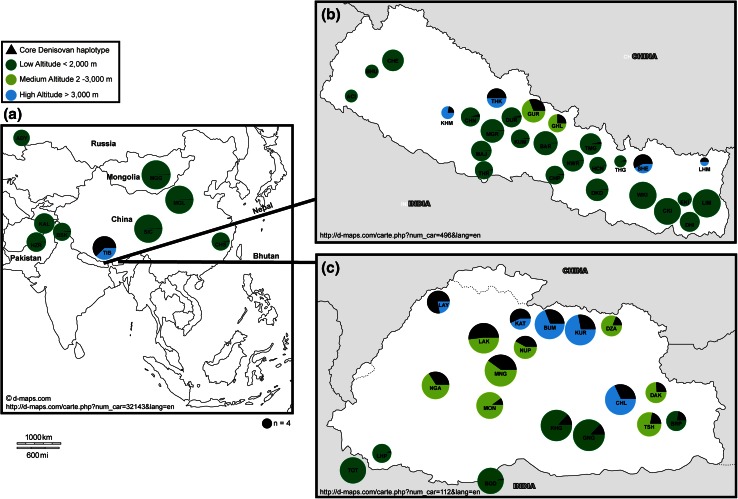
Core Denisovan haplotype frequency in **a** Eurasia, **b** Nepal and **c** Bhutan. *Circles* represent populations residing at various altitudes, indicated by *colours* as shown in the legend key. The *circle* areas are proportional to population sizes. Derived alleles for five SNPs (rs115321619, rs73926263, rs73926264, rs73926265, rs55981512) constitute the core Denisovan haplotype (AGGAA) and its frequency is shown as a *black pie-slice* inside each *coloured circle*. Three-letter population codes are defined in Table S1. The maps were obtained from d-map.com using the urls given in *each panel*

Derived alleles for the five SNPs (rs115321619, rs73926263, rs73926264, rs73926265, rs55981512) that constitute the core Denisovan haplotype (AGGAA) were present at a high frequency not only in Tibetans and Sherpa, but also among many ethno-linguistic groups from Bhutan and Nepal and nearby populations (Fig. 1). The core haplotype frequency was high (63 %) in the Tibetans analysed here, in line with previous reports (Huerta-Sanchez et al. 2014). We also confirmed the presence of the Denisovan AGGAA haplotype in the CHS individual from Phase I of the 1000 Genomes Project as reported earlier (The 1000 Genomes Project Consortium 2012).

We observed a differential distribution of the AGGAA haplotype across population groups in Bhutan and Nepal (Fig. 1). In Nepal, the core Denisovan haplotype was observed at frequency >25 % in all populations residing above altitudes of 2000 m. Except for the Kham (KHM), all high-altitude (>3000 m) populations from Nepal (Lohmi, Sherpa and Thakali) had a haplotype frequency >50 %. The haplotype was completely absent in Western and Central Kiranti, as well as in the Bahun (BHU), Chetri (CHE), Limbu (LIM) and the Indo-Aryan Artisanal Castes (ACI) who reside at low altitudes (Fig. 1). A similar pattern was seen in Bhutan where the highest frequency (78 %) of the AGGAA haplotype was observed in the Layap, who reside at 4115 m. In Bhutanese low-altitude populations, the haplotype frequency ranged from 6 to 29 %. The Denisovan haplotype was thus widespread in these populations, and was also observed in the Burusho (BSK; 11 % frequency) from Pakistan who reside in the Karakoram mountain valleys on the western fringe of the Himalayas at ~1000 m (Fig. 1), and at low (2 %) frequency in the Hazara (HZR) from Pakistan. Although both these Pakistani groups live at considerable distances from Tibet, they have documented East Asian admixture (Li et al. 2008). None of the Kalash (KAL), who reside in the Hindukush Mountains, or Adygei (ADY) individuals from the Caucasus carried the core Denisovan haplotype.

**Fig. 2:**
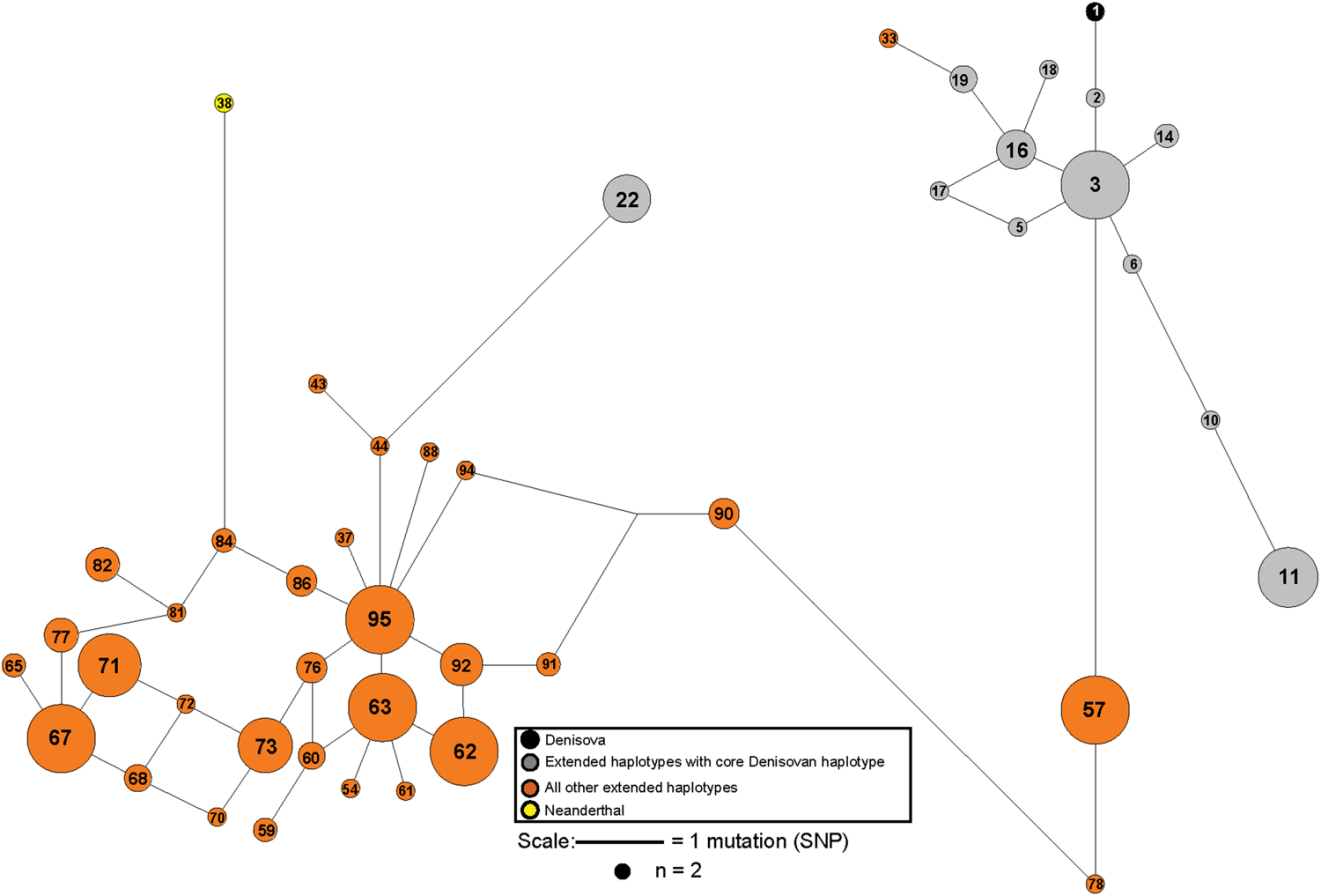
Median-joining network of genotyped intronic SNPs in *EPAS1.* The median-joining network was constructed using phased haplotypes of frequency >1. Each *coloured circle* represents a haplotype and its area is proportional to frequency. Archaic haplotypes are shown in *black* (Denisovan) and *yellow* (Neanderthal); *grey* *circles* represent chromosomes with an extended 19 SNP haplotype in which the five-SNP core Denisovan haplotype is present, whereas those in *orange* lack this five-SNP haplotype. *Lines* between haplotypes indicate mutational distance. The haplotypes are labelled as listed in Table S5. Haplotype 3 is common in Tibetans and is separated by six mutations from haplotype 57 that does not contain the core Denisovan haplotype

Phasing revealed 95 haplotypes (Tables S4 and S5) and a median-joining haplotype network shows two main haplotype clusters (orange and grey) separated by six SNPs (Fig. 2). A majority of the haplotypes carried by the high-altitude dwellers fall into a cluster that also includes the Denisovan individual. The core Denisovan AGGAA haplotype is present in multiple 19-SNP haplotypes found in all three Nepalese and Bhutanese altitude groups, as well as in Tibet and in one Burusho and one Southern Han individual, emphasizing its widespread prevalence throughout the Himalayan region. This implies that the introgression occurred in a common ancestral population and reached high frequency in populations residing at higher altitudes in the Himalayas, probably because it conferred a selective advantage. One haplotype carrying the core motif lies outside the main cluster and may have arisen via a recombination event.

### Correlation of the Denisovan haplotype with high altitude

**Fig. 3:**
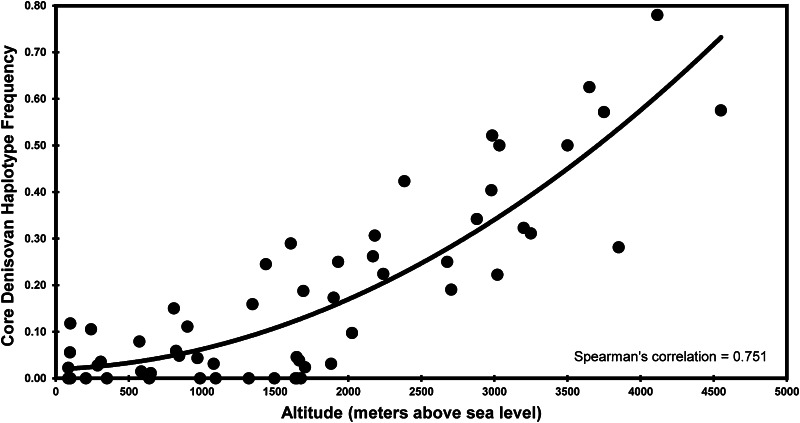
Correlation between altitude and core Denisovan haplotype frequency. Each *circle* represents a population sample genotyped in this study

By incorporating altitude information into our analyses, we were able to investigate the relationship between the frequency of this haplotype and altitude. We observed a very strong correlation (Fig. 3) between the frequency of the core Denisovan haplotype in the Himalayan populations and altitude of residence (Spearman’s correlation coefficient = 0.75, *p* value 3.9 × 10^−11^). This is among the highest genetic correlations reported for any trait. Previous studies have shown that the local pathogen diversity in the environment is the predominant driver of natural selection rather than climate, with correlations ranging between 0.40 and 0.61 (Fumagalli et al. 2009, 2011). The widespread prevalence of the introgressed haplotype in the Himalayas and its absence or low (<3 %) frequency in low-altitude populations in China support the earlier conclusion that the high-altitude adaptation might have occurred in a common ancestral population (Jeong et al. 2014), which might also have contributed to many of the other (especially high-altitude) populations in the area.

### Relationship of the selected haplotype to other sequence-based haplotypes

**Fig. 4:**
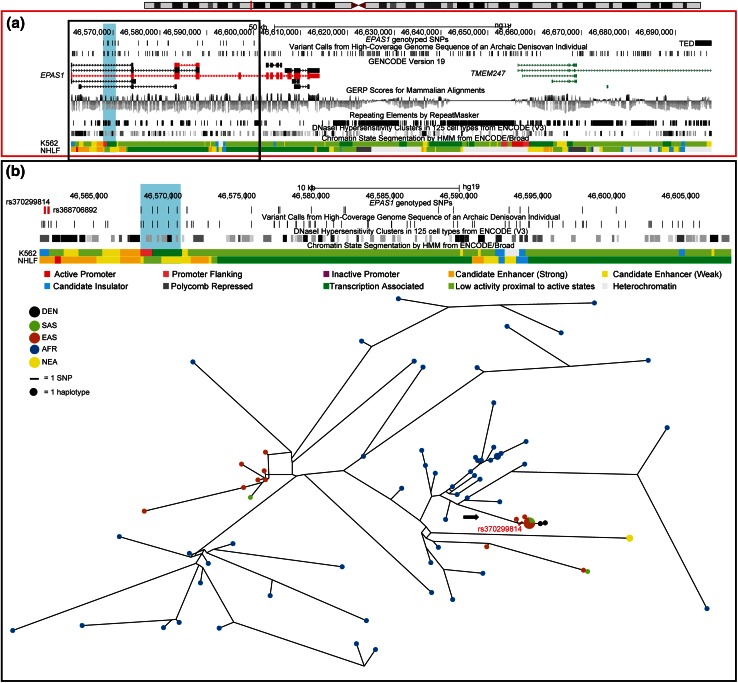
ENCODE regulatory features in region surrounding core Denisovan haplotype. **a** A 136,701-kb region on chromosome 2 (46,561,000–466,697,700 in GRCh37) that spans the *EPAS1* and *TMEM247* genes showing the SNPs genotyped in this study (*vertical lines*, *top row*), the copy number deletion frequent in Tibetans (TED block) and variants that differ between the human reference sequence (hg19) and the Denisovan, an archaic hominin. The region containing the five SNPs (rs115321619, rs73926263, rs73926264, rs73926265, rs55981512) that constitute the core Denisovan haplotype is highlighted in *blue*. The GENCODE (Version 19) transcript annotation is shown and the two *EPAS1* transcripts expressed in lung tissues are shown in *red*. The TED is located ~80-kb downstream in another gene (*TMEM247*) in a region that has low conservation GERP scores and no ENCODE-annotated regulatory feature in the human lung fibroblast cell line (NHLF). **b** An expanded view of the upstream ~40-kb region (demarcated by the *black box* in panel *a*). The *EPAS1* genotyped SNPs track shows the two plausible candidate regulatory variants (*vertical* *red lines*) that are located upstream of the core Denisovan haplotype (highlighted in *blue*). Both candidate variants are located in an evolutionarily conserved region of open chromatin, as depicted by the DNase I Hypersensitivity Clusters in the 125 cell line track, and show ENCODE chromatin state segmentation associated with an active promoter site in a human lung fibroblast cell line (NHLF). One of the SNPs (rs370299814) is associated with CTCF binding in many cells (like the chronic myelogenous leukemic K562 cell line shown here) and enhancer-1-associated marks in NHLF. The *lower part* of this panel shows a median-joining haplotype network for this region in the 1000 Genomes Project African and Asian samples, with *circles* representing haplotypes with areas proportional to frequency. Population origins are indicated by node *colours* as shown in the legend. Phased low-coverage sequences generated by Phase 3 of the 1000 Genomes Project were used for network construction along with the high-coverage Denisovan and Neanderthal sequences. Only African and Asian samples containing derived alleles for the core Denisovan haplotype were used in the network construction. The *arrow* indicates the branch leading towards the samples with the introgressed haplotype and the location of the candidate regulatory mutation (rs370299814) on this branch. Three mutations separate the common Asian high-altitude haplotype from the Denisovan, whereas the closest African haplotype is separated from this Asian haplotype by 22 mutations

We examined the derived allele frequency of the core Denisovan SNPs in the 1000 Genomes Project Phase 3 dataset, comprising 26 worldwide populations. Seven South- and East-Asian individuals shared the core Denisovan haplotype that extends beyond the 32.7-kb region. These additional samples consisted of two Bengali in Bangladesh (BEB), three Chinese Dai in Xishuangbanna, China (CDX) and two Kinh in Ho Chi Minh City, Vietnam (KHV), further attesting to the presence of this haplotype at low frequencies over a vast geographic area. We also observed derived alleles for all five SNPs that characterize the core Denisovan haplotype in all African populations, as reported earlier (Huerta-Sanchez et al. 2014) with frequencies ranging from 3 to 14 %. A median-joining network incorporating the African samples with derived alleles for these variants along with the additional Asian samples shows that only three mutations separate the Denisovan from the most frequent Asian haplotype, whereas the closest African haplotype is separated from it by 22 mutations (Fig. 4). The presence of these SNPs in Africans could represent incomplete lineage sorting of variants present in a population ancestral to Denisovans and modern humans, and thus does not conflict with the hypothesis of Denisovan introgression. However, one of the mutations that separates the Denisovan haplotype from the common high-altitude Tibetan haplotype is rs150877473, which is associated with low haemoglobin levels in Tibetans. The Denisovan sample has the ancestral C allele, whereas the Tibetans and other high-altitude populations have a higher frequency of the derived G allele than low-altitude populations suggesting that selection occurred on the Denisovan-related haplotype.

### Model of positive selection in *EPAS1* in high-altitude populations

The model-based SelEstim method highlighted the presence of three regions in *EPAS1* (named Region 1, Region 2, and Region 3) characterized by markers with different scaled selection coefficients; in particular, there are two regions within the ~32-kb introgressed *EPAS1* intron with high scaled selection coefficients (Fig. S1). Region 1, which lies between rs115321619 and rs149306391 and includes the core Denisovan haplotype, shows the highest scaled selection coefficients in high-altitude dwellers (Tibetans = 118, KAT = 113, SHE = 108). The selection coefficients are low in all low-altitude populations examined (~1). The middle section (Region 2) does not appear to be under positive selection in any population examined here and lies between variants rs4953354 and rs7586141. The downstream region (Region 3) also shows a high selection coefficient corresponding to rs150877473, although the mean selection coefficient of this region is lower than that of Region 1 (Fig. S1). The derived G allele of this variant is present at high frequency in Tibetans and is reported to be associated with low haemoglobin levels that characterize the high-altitude adaptation response in this population (Yi et al. 2010). However, the Denisovan haplotype carries the ancestral allele for this variant. Association analyses with altitude seemed to follow this pattern showing three regions with the highest −log_10_*p* values for Regions 1 and 3, corresponding to the SelEstim modelling. Interestingly it seems that the additive model is the one with the highest −log_10_*p* value suggesting that rs150877473, which is absent in Africa, may have arisen on the haplotype that introgressed after its split from the sequenced Denisovan individual and was swept to high frequency because it was beneficial to high-altitude adaptation. The haplotype structure based on mean derived allele frequencies also supports the hypothesis of three different regions in *EPAS1*, with different levels of selection pressure acting on them (Fig. S2). Combining the results of all these three analyses, we suggest that selection on *EPAS1* is driven by variants in Region 1 that form the core Denisovan haplotype, which show the SNPs with the highest scaled selection coefficients, and that these may be interacting with rs150877473 in an additive manner. Simulations with simuPOP v 1.1.6 (Peng and Kimmel 2005) using the highest scaled selection coefficient (*σ* = 2 × *N*_e_ × *s*) in Tibetans and three published demographic models (Qi et al. 2013; Yi et al. 2010) show that the high derived allele frequency of *EPAS1* SNPs in Tibetans can be explained by positive selection acting on a functional variant that is present at an initial frequency between 0.05 and 0.1 (Fig. S3a) ~2800 years ago, assuming a generation time of 30 years. This is similar to the observed frequency of the core Denisovan haplotype in many low-altitude populations from Bhutan and Nepal. If we assume that the Tibetans and Han split during the Palaeolithic (~25,000 years ago) then simulations predict that after 1000 generations the derived allele should become fixed, which implies that in this scenario the selection must have occurred much later. The same conclusion is obtained from the scenario in which the split between Tibetans and Han happened 10,000 years ago (Fig. S3b–c). Thus, it seems likely that selection at this locus occurred between 2800 and 10,000 years ago. However, more clarity about the demographic history of Tibetan populations and their admixture patterns is needed to effectively model the evolutionary history of genes linked to high altitude in these populations.

### Identifying candidate functional variants in *EPAS1* associated with high altitude

The five SNPs comprising the core Denisovan haplotype studied here span a region of 2.4-kb. However, none of these SNPs have been previously associated with a specific adaptive trait, and it is possible that the functional variant responsible for the adaptive phenotype in Tibetans and other high-altitude Himalayan populations is simply tagged by the core haplotype. A recent study implicated a 3.4-kb deletion ~80.4-kb downstream from *EPAS1* that was enriched in Tibetans and in complete LD with the core Denisovan haplotype and other variants associated with the reduced haemoglobin Tibetan phenotype (Lou et al. 2015). However, this deletion does not lie in an Encyclopedia of DNA Elements (ENCODE)-annotated regulatory region in the lung fibroblast (NHLF) cell line (Fig. 4), a proxy for the lung tissue in which *EPAS1* is highly expressed (The GTEx Consortium 2015).

The presence of the introgressed haplotype in several additional 1000 Genomes Project samples from East and South Asia, including the two Han Chinese identified earlier (Huerta-Sanchez et al. 2014), allowed us to use the sequence data to refine the haplotype structure and identify putative functional candidates. As expected, the length of the introgressed segment was variable in these individuals and in some cases much longer than 32 kb. These Asian samples shared derived alleles with the archaic haplotype for ~40-kb, extending ~7 kb upstream of the 32.7-kb region reported initially. In this upstream region there are thirteen intronic SNPs in the 1000 Genomes Project samples. Two of these (rs370299814 and rs368706892) share the same pattern of being derived, present in the Denisovan genome, and, within the 1000 Genomes Project, specific to the samples that contain the high-altitude Denisovan haplotype. The other eleven SNPs are more common in the 1000 Genomes Project samples and thus not informative, although all samples that contain the core Denisovan haplotype share the Denisovan allele for these SNPs. These 11 variants are either ancestral (9) or derived (2) in the Denisovan. Functional annotation of the derived alleles in these samples that were shared with the high-coverage Denisovan sequence indicated that both rs370299814 and rs368706892 were plausible candidate regulatory variants. Both variants lie in the *EPAS1* region that has been identified by the ENCODE Consortium as being promoter-flanking in the NHLF cell line (Fig. 4). These variants lie upstream of the major *EPAS1* transcript in the lungs (ENST00000466465) and within the intron of another transcript (ENST00000263734). These two transcripts are the only *EPAS1* transcripts (out of ten annotated by GENCODE, version 19) that are expressed in the lung tissue according to the GTEx Analysis Release V4 data (The GTEx Consortium 2015).

One of the candidate regulatory variants (rs370299814) is associated with CTCF binding in all cell lines except NHLF. Although the derived allele for this variant does not disrupt the consensus binding motif for CTCF, it nevertheless could affect CTCF binding, possibly leading to modified *EPAS1* gene expression that could contribute to the high-altitude hypoxic response in these Himalayan populations. If there is a decrease in the expression of *EPAS1* in lung tissue, this could explain the reduced haemoglobin and ventilatory responses and protection from pulmonary hypertension documented in Tibetans and Sherpa in response to hypoxia (Bigham and Lee 2014). Functional follow-up is needed to provide further evidence for this model.

## Conclusions

We show that the Denisovan *EPAS1* haplotype is present in many populations from Bhutan, Nepal and nearby, and that the 5-SNP core Denisovan haplotype frequency is strongly correlated with altitude. The presence of the introgressed haplotype at low frequencies in populations as far apart as Bangladesh and Vietnam shows that the haplotype is widespread in Asia and suggests that the Denisovan introgression occurred in a population ancestral to many of the Himalayan highlanders. Variants that characterize the core Denisovan haplotype appear to be interacting with other variants (such as rs150877473) in an additive manner, and the key functional selected variants may affect *EPAS1* expression in lung tissue. Future work could aim to combine gene expression and functional assays to unravel the mechanism of action of the *EPAS1* candidate regulatory SNPs.

## Electronic supplementary material

Supplementary material 1 (TIFF 1016 kb)

Supplementary material 2 (TIFF 1193 kb)

Supplementary material 3 (TIFF 326 kb)

Supplementary material 4 (DOCX 25 kb)

Supplementary material 5 (XLSX 12 kb)

Supplementary material 6 (XLSX 185 kb)

Supplementary material 7 (XLSX 485 kb)

Supplementary material 8 (XLSX 16 kb)
