# Supplementary figures and images for: Wide distribution and altitude correlation of an archaic high-altitude-adaptive EPAS1 haplotype in the Himalayas
Source: Hum Genet. 2016 Feb 16;135:393–402. doi: 10.1007/s00439-016-1641-2 (PMC4796332; doi:10.1007/s00439-016-1641-2)

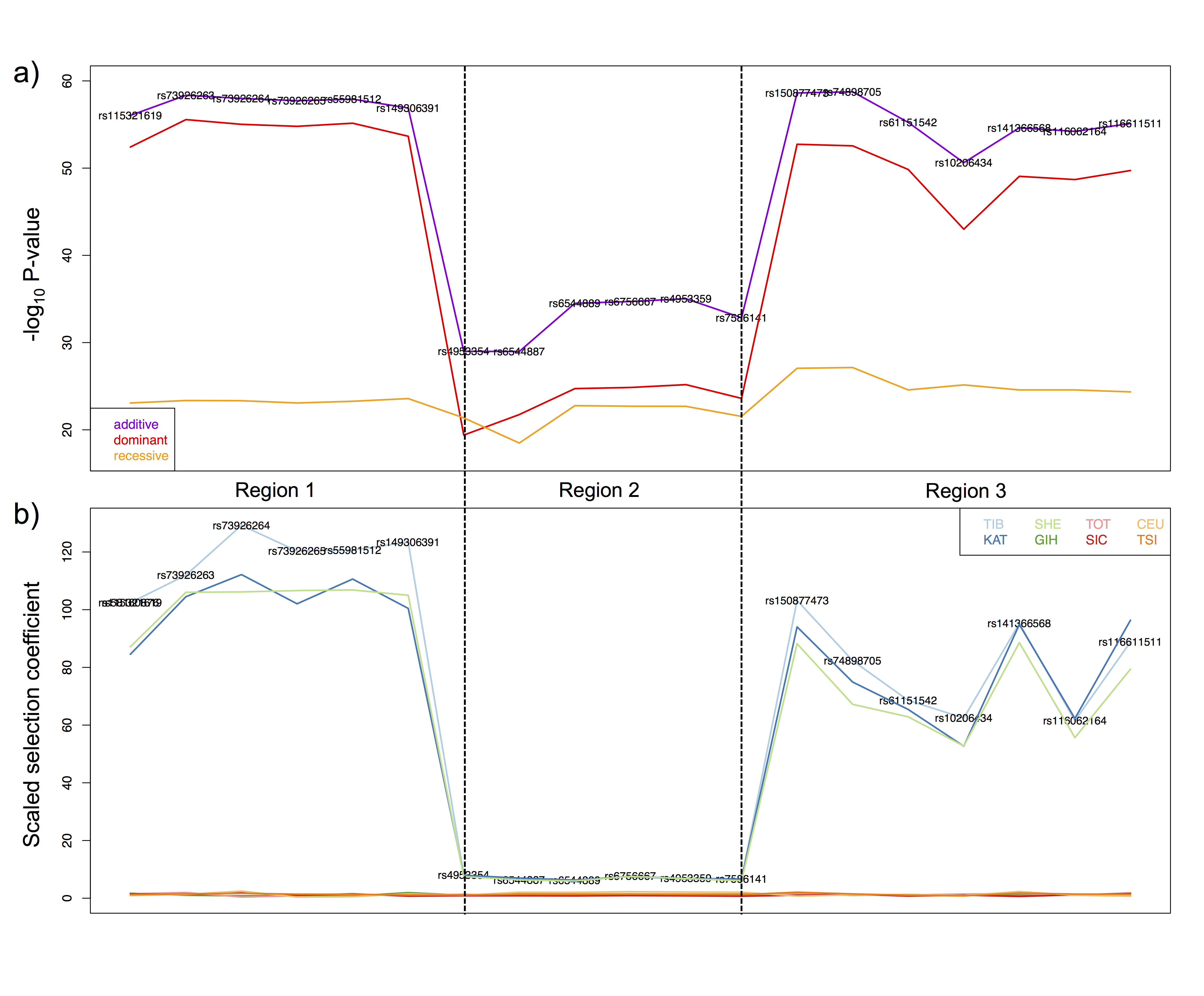

Supplement: Supplementary file 1 — Supplementary material 1 (TIFF 1016 kb) [file 439_2016_1641_MOESM1_ESM.tif]

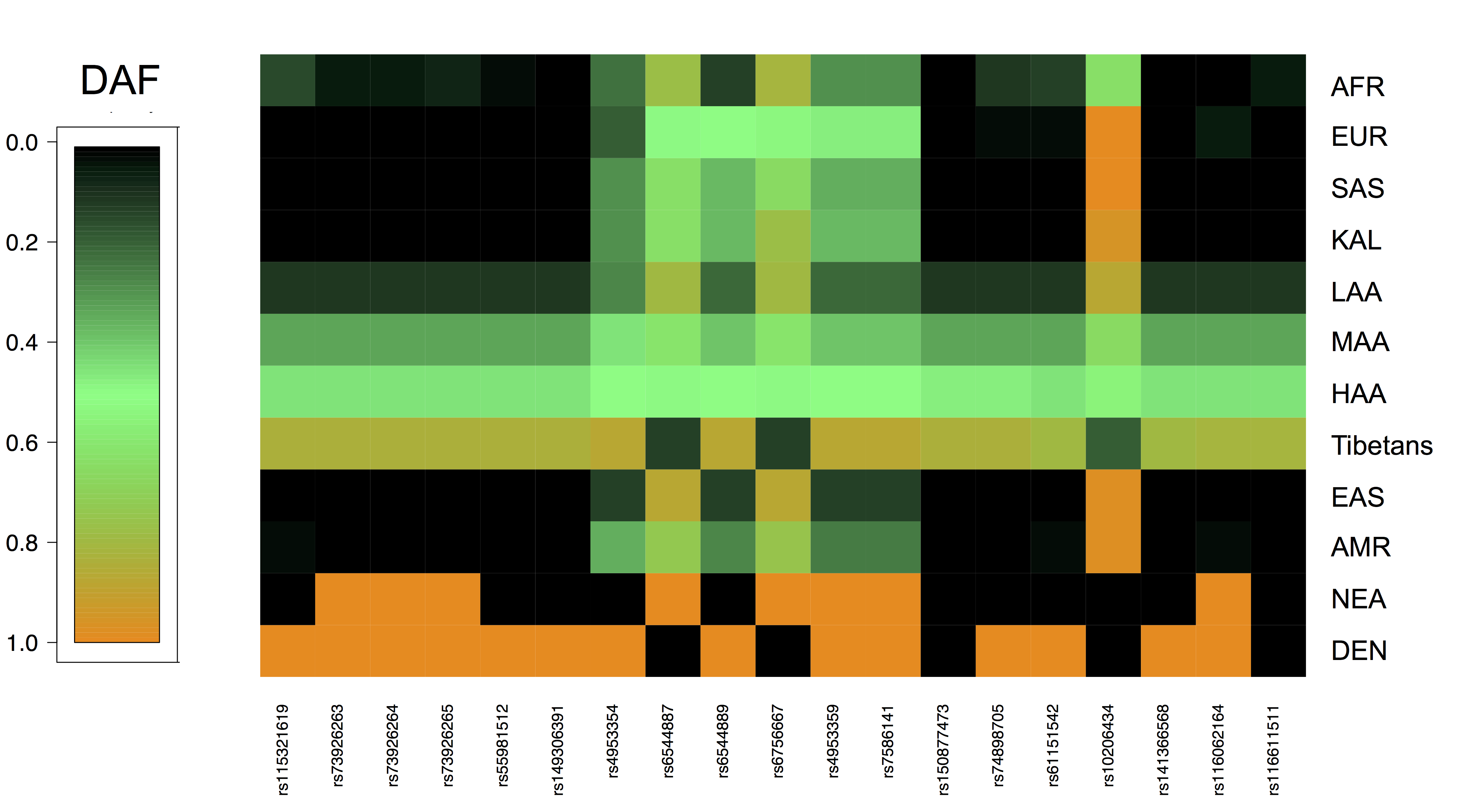

Supplement: Supplementary file 2 — Supplementary material 2 (TIFF 1193 kb) [file 439_2016_1641_MOESM2_ESM.tif]

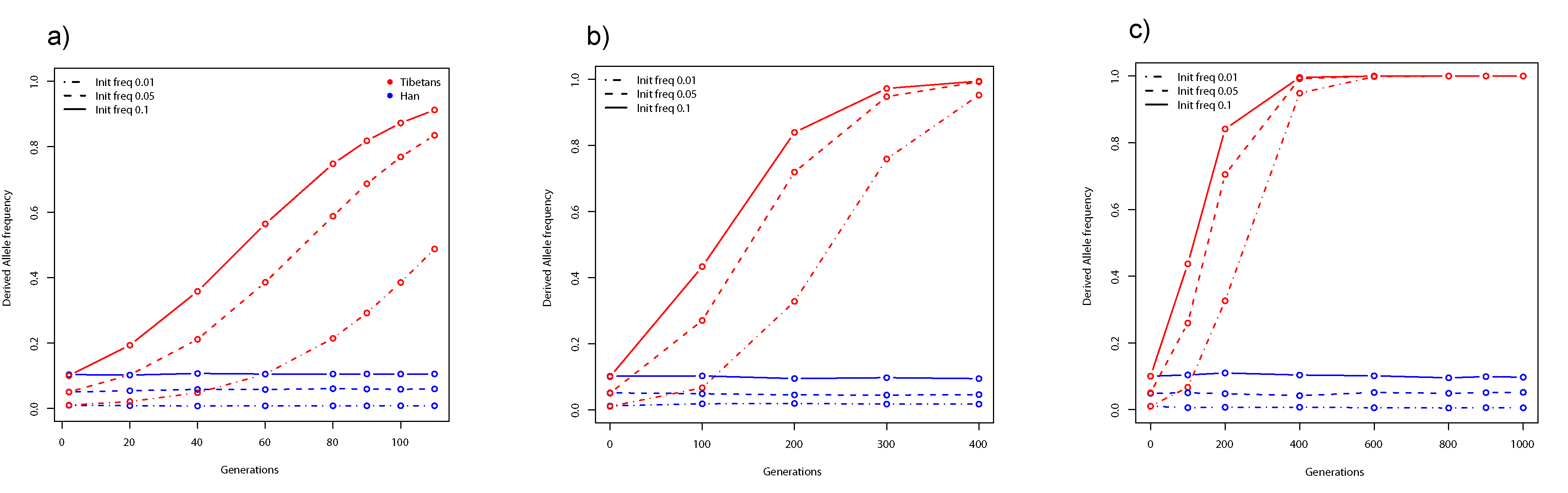

Supplement: Supplementary file 3 — Supplementary material 3 (TIFF 326 kb) [file 439_2016_1641_MOESM3_ESM.tif]
