# Supplementary material for: Wide distribution and altitude correlation of an archaic high-altitude-adaptive EPAS1 haplotype in the Himalayas
Source: Hum Genet. 2016 Feb 16;135:393–402. doi: 10.1007/s00439-016-1641-2 (PMC4796332; doi:10.1007/s00439-016-1641-2)
Supplement: Supplementary file 4 — Supplementary material 4 (DOCX 25 kb) [file 439_2016_1641_MOESM4_ESM.docx]

**Supplementary Table 1. Analyzed populations and samples.**

List of ethno-linguistic genotyped populations from Bhutan, China, India, Mongolia, Nepal, Pakistan and Russia. Sample size indicates the number of individuals for whom genotyping data was available after quality control. Altitude corresponds to the elevation (m = meters above sea level) at which each group resides.

| **Population** | **Code** | **Language** | **Sample Size** | **Altitude (m)** |
| --- | --- | --- | --- | --- |
| **Bhutan (n= 594)** | | | | |
| Black Mountain Mönpa | MON | Tibeto-Burman | 36 | 2027 |
| Brokkat | KAT | Tibeto-Burman | 21 | 3750 |
| Brokpa | BRP | Tibeto-Burman | 19 | 1608 |
| Bumthang | BUM | Tibeto-Burman | 45 | 3250 |
| Chali | CHL | Tibeto-Burman | 48 | 3201 |
| Dakpa | DAK | Tibeto-Burman | 21 | 2169 |
| Dzala | DZA | Tibeto-Burman | 21 | 2705 |
| Dzongka (‘Ngalop) | NGA | Tibeto-Burman | 38 | 2880 |
| Gongduk | GNG | Tibeto-Burman | 51 | 1437 |
| Khengpa | KHG | Tibeto-Burman | 50 | 808 |
| Kurtöp | KUR | Tibeto-Burman | 48 | 3850 |
| Lakha | LAK | Tibeto-Burman | 47 | 2985 |
| Layap | LAY | Tibeto-Burman | 25 | 4115 |
| Lhokpu | LHP | Tibeto-Burman | 17 | 823 |
| Mangde | MNG | Tibeto-Burman | 52 | 2980 |
| Nup | NUP | Tibeto-Burman | 26 | 2385 |
| Tshangla (Shâchop) | TSH | Tibeto-Burman | 29 | 2240 |
| **China (n = 127)** | | | | |
| Inner Mongolia | MGL | Altaic | 44 | 650 |
| Sichuan | SIC | Sino-Tibetan | 34 | 450-720 |
| Southern Han Chinese | CHS | Sino-Tibetan | 18 | 286 |
| Tibet | TIB | Tibeto-Burman | 31 | 3650 |
| **India (n = 68)** | | | | |
| Bodo | BOD | Tibeto-Burman | 34 | 99 |
| Toto | TOT | Tibeto-Burman | 34 | 86 |
| **Mongolia (n = 47)** | | | | |
| Mongolian | MGG | Altaic | 47 | 650 |

| **Population** | **Code** | **Language** | **Sample Size** | **Altitude (m)** |
| --- | --- | --- | --- | --- |
| **Nepal (n= 594)** | | | | |
| Bahun (Brahmin) | BHU | Indo-European | 10 | 1320 |
| Baram | BAR | Tibeto-Burman | 32 | 1080 |
| Central Kiranti | CKI | Tibeto-Burman | 40 | 640 |
| Chantyal | CHN | Tibeto-Burman | 20 | 1932 |
| Chepang (Praja) | CHP | Tibeto-Burman | 19 | 241 |
| Chetri (Kshetriya) | CHE | Indo-European | 26 | 987 |
| Dhimal | DHI | Tibeto-Burman | 22 | 86 |
| Dura | DUR | Tibeto-Burman | 22 | 1346 |
| Eastern Kiranti | EKI | Tibeto-Burman | 11 | 1648 |
| Ghale | GHL | Tibeto-Burman | 18 | 2678 |
| Gurung | GUR | Tibeto-Burman | 31 | 2182 |
| High Caste Newar | HCN | Tibeto-Burman | 16 | 1883 |
| Indo-Aryan Artisanal Caste | ACI | Indo-European | 9 | 206 |
| Danuwar | DKD | Indo-European | 28 | 308 |
| Kham (Magar) | KHM | Tibeto-Burman | 9 | 3021 |
| Kumal | KUM | Indo-European | 19 | 572 |
| Lhomi | LHM | Tibeto-Burman | 4 | 3500 |
| Limbu | LIM | Tibeto-Burman | 44 | 1094 |
| Magar | MGR | Tibeto-Burman | 31 | 844 |
| Majhi (Bote) | MAJ | Indo-European | 23 | 969 |
| Newar | NWR | Tibeto-Burman | 26 | 1665 |
| Sherpa (Solu-Khumbu) | SHE | Tibeto-Burman | 20 | 4550 |
| Tamang | TMG | Tibeto-Burman | 26 | 1900 |
| Thakali | THK | Tibeto-Burman | 21 | 3035 |
| Thangmi | THG | Tibeto-Burman | 8 | 1693 |
| Tharu | THR | Indo-European | 18 | 97 |
| Western Kiranti | WKI | Tibeto-Burman | 41 | 1496 |
| **Pakistan (n = 62)** | | | | |
| Burusho | BSK | Isolate | 18 | 900 |
| Hazara | HZR | Indo-European | 21 | 1705 |
| Kalash | KAL | Indo-European | 23 | 1640 |
| **Russia (n = 15)** | | | | |
| Adygei | ADY | North Caucasian | 15 | 1676 |
